# Scrapie-Specific Pathology of Sheep Lymphoid Tissues

**DOI:** 10.1371/journal.pone.0001304

**Published:** 2007-12-12

**Authors:** Gillian McGovern, Martin Jeffrey

**Affiliations:** Veterinary Laboratories Agency (VLA) Lasswade, Pentlands Science Park, Bush Loan, Penicuik, Midlothian, United Kingdom; University of Liverpool, United Kingdom

## Abstract

Transmissible spongiform encephalopathies (TSEs) or prion diseases often result in accumulation of disease-associated PrP (PrP^d^) in the lymphoreticular system (LRS), specifically in association with follicular dendritic cells (FDCs) and tingible body macrophages (TBMs) of secondary follicles. We studied the effects of sheep scrapie on lymphoid tissue in tonsils and lymph nodes by light and electron microscopy. FDCs of sheep were grouped according to morphology as immature, mature or regressing. Scrapie was associated with FDC dendrite hypertrophy and electron dense deposit or vesicles. PrP^d^ was located using immunogold labelling at the plasmalemma of FDC dendrites and, infrequently, mature B cells. Abnormal electron dense deposits surrounding FDC dendrites were identified as immunoglobulins suggesting that excess immune complexes are retained and are indicative of an FDC dysfunction. Within scrapie-affected lymph nodes, macrophages outside the follicle and a proportion of germinal centre TBMs accumulated PrP^d^ within endosomes and lysosomes. In addition, TBMs showed PrP^d^ in association with the cell membrane, non-coated pits and vesicles, and also with discrete, large and random endoplasmic reticulum networks, which co-localised with ubiquitin. These observations suggest that PrP^d^ is internalised via the caveolin-mediated pathway, and causes an abnormal disease-related alteration in endoplasmic reticulum structure. In contrast to current dogma, this study shows that sheep scrapie is associated with cytopathology of germinal centres, which we attribute to abnormal antigen complex trapping by FDCs and abnormal endocytic events in TBMs. The nature of the sub-cellular changes in FDCs and TBMs differs from those of scrapie infected neurones and glial cells suggesting that different PrP^d^/cell membrane interactions occur in different cell types.

## Introduction

Scrapie, a disease naturally affecting British sheep and goats for many years, belongs to a group of slowly progressive neurodegenerative disorders, the transmissible spongiform encephalopathies (TSEs) or prion diseases, which include infectious, familial and sporadic forms of disease in animals and man. The TSEs include bovine spongiform encephalopathy (BSE), scrapie of sheep and goats, Creutzfeldt-Jakob disease (CJD), kuru and Gertsmann-Straűssler syndrome (GSS) of humans. TSEs result in abnormal isoforms of a host-coded, cell-surface glycoprotein called prion protein (PrP).

Unlike the normal proteinase sensitive form of prion protein (PrP^sen^) abnormal PrP detected by immunoblotting methods is abnormally resistant to proteinase treatment and contains truncated, protease resistant forms of PrP often designated PrP^res^ [1]. While these biophysically altered forms of PrP are a reliable markers for the presence of infectivity, not all infectious preparations contain PrP^res^. Immunohistochemistry may also be used to detect disease-associated accumulations of PrP (PrP^d^). Unlike immunoblotting methods, immunohistochemistry detects abnormal PrP forms that may be truncated or full length, protease resistant or protease sensitive [2], often designated PrP^d^.

PrP^d^ accumulates in the nervous system and lymphoreticular system (LRS) scrapie of sheep and variant Creutzfeldt Jakob disease. In contrast with the CNS, where lesions are well known, it is commonly accepted that despite the presence of infectivity and PrP^d^ accumulation, there is no associated pathology in the lymphoreticular system of naturally occurring TSEs. Studies using severe combined immunodeficient mice and chimaeric mice indicate that follicular dendritic cells (FDCs) are necessary for prion propagation within the LRS [3]. In addition to FDCs, macrophages of the LRS have been identified as reservoirs of the TSE infectious agent [4]. Tingible body macrophages (TBMs), so named due to their dark-staining, phagocytosed nuclear remnants in their cytoplasmic vesicles, are normal constituents of the germinal centres of secondary lymphoid tissues [5], and contain abundant PrP^d^ as demonstrated by immunohistochemistry in scrapie [6] and vCJD [7]. The exact mechanism by which infection reaches lymphoid follicles and FDCs remains unclear. However, FDCs are responsible for the trapping and retention of antigens in association with antibodies on their cell surface. This trapping is initiated by the interactions of complement and cellular complement receptors, and antibodies and their complementary receptors on the FDC plasmalemma [8].

Within affected lymph nodes of scrapie-affected sheep, most secondary follicles show PrP^d^ accumulation [9], [10]. Normal gut-associated lymphoid tissue (GALT) development is known to be related to age with Peyer's patches of young sheep constituting a major component of GALT. With the exception of the tonsil, GALT of sheep undergoes progressive involution at around the time of sexual maturity [11], [12], [13]. However, this involution may be delayed in scrapie-affected sheep [14], which might provisionally indicate a scrapie-related pathology in the LRS.

Labelling of CD21, which is expressed on FDC membranes and on B cells [15], co-localises with PrP^d^ immunolabelling only on cells morphologically similar to mature FDCs in the light zone of germinal centres of secondary follicles. In contrast, TBM labelling is present in the light, dark, mantle and paracortical zones [6]. PrP^d^ labelling has also been detected within mononuclear cells of the periarteriolar lymphoid sheath (PALS) and within the marginal zone of the spleen [16]. Previous studies of TSE-affected sheep and mice have demonstrated that intracellular PrP^d^ accumulations are truncated with the loss of the N-terminus amino acid sequence from approximately 23–90, while all other types of PrP^d^ accumulation remain full length [6], [2], [17]. Sub-cellular morphological studies of spleens from mice terminally affected by scrapie, demonstrate that FDCs form abnormally convoluted labyrinthine structures, and irregular, excess electron-dense deposits associated with dendrites [18], [19]. Immunogold labelling for PrP^d^ is mostly associated with the FDC dendrite plasmalemma and FDC dendrites engaged in plasmablast emperipolesis (i.e. FDC dendrite-encircled plasmablasts). PrP^d^ is also present in TBM lysosomes. The absence of the N-terminus of intra-cellular PrP^d^ in TBMs is consistent with the notion that TBMs internalize and digest extra-cellular PrP^d^ derived from FDCs [20].

In previous studies we have suggested that there are pathological changes associated with scrapie infection of splenic secondary follicles. To determine whether similar changes are present in natural disease, we have studied the morphological response and sub-cellular location of PrP^d^ in scrapie-infected sheep LRS tissues.

## Results

### Light Microscopy–wax

**Figure 1:**
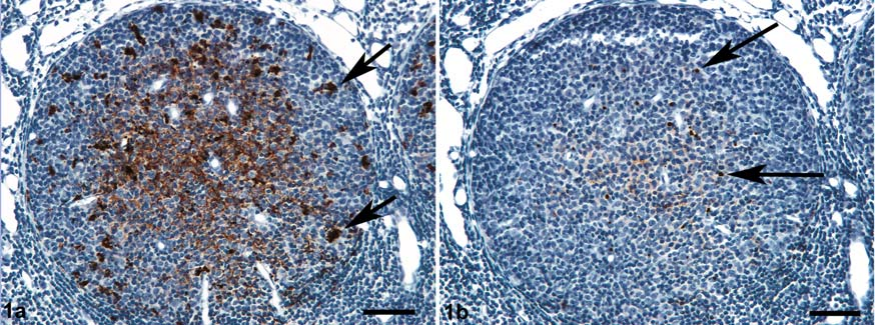
Immunolabelling of scrapie-affected tonsil using antibodies directed to the C' or N' terminus of PrP. Paraffin-embedded tonsil from clinically-affected sheep. Serial sections. PrP^d^ immunolabelling. a. The follicle shows both widespread follicular dendritic cell (FDC) labelling interspersed between lymphocytes and multiple granules of intracytoplasmic tingible body macrophage (TBM) labelling (arrows) using R145 antibody. Bar, 130 µm. b. Using BG4 antibody, light FDC labelling and single intracytoplasmic granules of TBM labelling are present (arrows). Bar, 130 µm.

**Figure 2:**
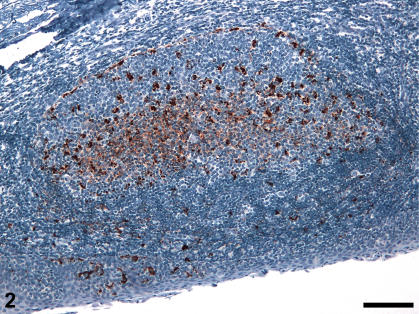
PrP^d^ immunolabelling of follicle and perifollicular area of scrapie-affected tonsil. Paraffin-embedded tonsil from clinically-affected sheep. PrP^d^ immunolabelling. Diffuse follicular dendritic cell (FDC) and granular tingible body macrophage (TBM) labelling are present within the follicle, additionally, granular labelling is associated with the peri-follicular area and the epithelium of the tonsil. Follicular dendritic cell labelling is present throughout the light zone of the follicle (bottom) while TBM labelling is conspicuous in the dark zone (top) Bar, 290 µm.

Consistent with previous results [10], [14], [20] serial sections of lymph nodes and tonsil germinal centres labelled with the N terminal antibody BG4 or the C terminal antibody R145 were found to show similar patterns of FDC-type labelling in all LRS tissues. In contrast, marked differences in TBM labelling patterns were found for these two antibodies (Fig 1). Antibodies to the C terminus of the molecule gave an abundant intracellular pattern of labelling with multiple granules present within each macrophage. Using BG4, single granules were observed within the cytoplasm of a proportion of TBMs within the light zone and to a lesser extent the dark zone of the follicle. These findings were consistent with those seen in different natural and experimental sheep scrapie strains and sources [20]. Using R145, abundant PrP^d^ labelling was observed within macrophages of the perifollicular region of the mesenteric and submandibular lymph nodes. Within tonsils, PrP^d^ was associated with macrophages present in thinner regions of the epithelial layer, and in the perifollicular area. This labelling was not revealed by BG4 antibody (Fig 2).

### Light Microscopy–resin

Secondary follicles from scrapie-affected sheep showed PrP^d^ labelling mainly within germinal centres, and the granular intracellular labelling observed within the perifollicular area of the tonsil and the medulla of the lymph nodes in paraffin wax embedded sections, could also be visualised in resin embedded tissues. PrP^d^ labelling within germinal centres of the secondary follicle was consistent with a linear FDC-type pattern, and a multi-granular intracellular TBM accumulation of PrP^d^ as previously described [18]. Multiple mature secondary follicles (between 2 and 8 per tissue block), each with an extensive light zone containing mature FDCs were identified in sections from studied tissue blocks (n = 60) from scrapie-affected lymph nodes. Fewer secondary follicles (between 1 and 4 per tissue block), none of which were PrP^d^ positive, were observed in lymph node blocks (n = 16) from unaffected animals. The intensity of PrP^d^ immunolabelling in scrapie affected animals varied between follicles, tissue blocks and animals, although most follicles showed extensive PrP^d^ accumulations, primarily within the follicle.

**Figure 3:**
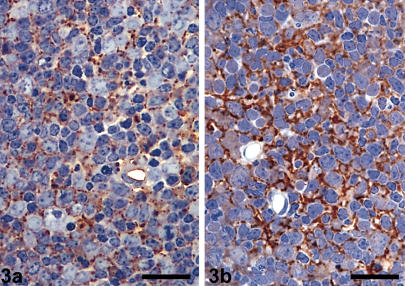
Immunoglobulin in normal and scrapie-affected follicles-light microscopy. a. Resin embedded Submandibular lymph node from an uninfected sheep. Immunoglobulin labelling. Immunolabelling is interspersed between lymphocytes of the germinal centre as branching networks, consistent with follicular dendritic cell (FDC)-associated labelling. Bar, 55 µm. b. Resin embedded Submandibular lymph node from clinically-affected sheep. Immunoglobulin labelling. A similar yet considerably more intense pattern of immunoglobulin labelling is present between lymphocytes of the germinal centre. Accumulations appear more expanded within the extracellular space when compared with the uninfected control shown in figure 3a. Bar, 55 µm.

In uninfected animals, low levels of immunoglobulin (IgG) labelling were observed interspersed in a curvi-linear pattern between lymphocytes of the germinal centre (Fig 3a). A similar but considerably more intense pattern of labelling was observed in the follicles of scrapie-affected animals. The pattern of labelling corresponded with an FDC PrP^d^ pattern (Fig 3b).

Following application of an anti-ubiquitin antibody, a few granules of immunolabelling were identified within cells morphologically similar to TBMs in scrapie-affected tonsils or lymph nodes.

### Electron Microscopy (EM)

#### Morphology and immunolabelling of uninfected ovine lymphoid tissues

**Figure 4:**
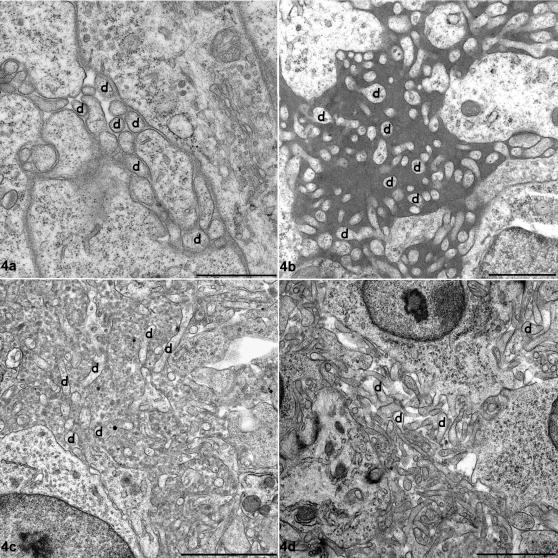
Ultrastructural follicular dendritic cell (FDC) morphology. Uranyl acetate (UA)/lead citrate (LC) counterstain. a. Immature FDC dendritic processes from an unaffected sheep submandibular lymph node (SMLN). Simple FDC processes run between lymphocytes, a number of which are labelled (d). The extracellular space surrounding FDC dendrites is uniform in width and contains no electron dense deposit. Bar, 1 µm. b. Disease-associated mature type 1 FDC dendritic processes from a scrapie-affected sheep SMLN. FDC processes are more numerous and convoluted with abundant uniformly-electron dense deposit within the expanded extracellular space. Again, a selection of dendrites are labelled (d). Bar, 1 µm. c. Disease-associated mature type 2 FDC dendritic processes from a scrapie-affected sheep SMLN. Dendrites are convoluted and the associated extracellular space grossly expanded. The space contains many indistinct spherical or ovoid structures. Electron dense deposit as seen in figure 4b is absent from this space. Selected dendrites are labelled (d). Bar, 2 µm. d. Regressing FDC from an unaffected sheep. Dendrites form distinct rod-like projections. Electron dense deposit and vesicular structures are limited or absent while the extracellular space is clearly expanded. Selected dendrites are labelled (d). Bar, 2 µm.

**Figure 5:**
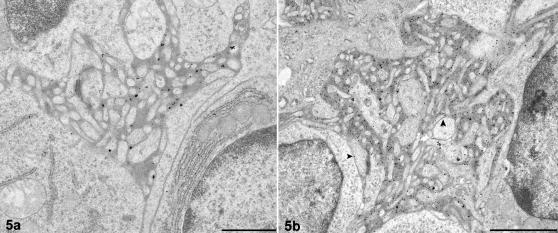
Immunoglobulin in normal and scrapie-affected follicles-electron microscopy. Follicular dendritic cell (FDC) dendrite immunoglobulin retention. a. FDC processes from an unaffected sheep tonsil. IgG immunogold labelling. Limited immunogold labelling is associated with the electron dense deposit between dendritic processes of a mature FDC. Occasionally, immunogold is located on the plasmalemma of these processes. Bar, 1 µm. b. FDC processes from a scrapie-affected sheep tonsil. IgG immunogold labelling. Immunogold labelling is primarily associated with the extensive electron dense deposit (Note difference in scale from control tissue in 5a) between processes of the disease-associated mature type 1 FDC. Where electron dense deposit is less abundant, immunogold can be identified on the plasmalemma of FDC dendrites (arrowheads). Bar, 2 µm.

Groups of FDC dendrites, morphologically similar to those found in normal rodents [18], were present within the light zone of the germinal centres of control tissues. Dendritic branches varied in complexity suggesting different stages of maturation. To simplify description, FDCs have been classified as immature, mature or regressing. The majority of immature FDC dendrites formed small knots between lymphocytes, with a uniform space between them. No electron dense deposit or vesicular structures accumulated in the extracellular space surrounding these dendrites (Fig 4). Mature FDCs were also identified within the light zones of follicles from uninfected sheep. These FDCs were only present within larger germinal centres of the follicles, which also contained many TBMs, and their dendrites were more florid, with small pockets of accumulated uniform electron dense deposits between adjacent dendritic profiles. A limited number of regressing FDCs were also present, again only within the larger germinal centres (Fig 4). Their dendrites appeared distinct and more linear, with limited electron dense deposit interspersed within the extracellular space between dendrites, occasionally, a few vesicular structures could be seen within this space. Limited immunoglobulin labelling was only observed in association with the FDC dendritic plasmalemma and the electron dense deposit held between developed mature type FDC dendrites (Fig 5).

TBMs containing lysosomes were also present in the light zone and dark zone of all follicles. Occasionally, TBMs contained whole apoptotic lymphocytes. Neither PrP^d^ nor ubiquitin labelling was observed in any sections studied.

#### Morphology and immunolabelling of scrapie affected ovine lymphoid tissues

Some normal mature FDCs were occasionally seen within scrapie-affected lymph nodes, but most FDCs showed abnormal morphologic changes of two types, which we categorised as disease-associated mature type 1 or type 2 FDCs. When compared with normal mature FDCs, disease-associated mature type 1 FDCs had more abundant florid dendrites with an excess accumulation of uniform electron dense deposit surrounding them (Fig 4b). These dendrites formed intense foci of interwoven dendrites or labyrinthine complexes. Disease-associated mature type 2 FDCs were also identified within highly reactive scrapie-affected germinal centres. This cell type had proliferative abnormalities of dendritic process similar to those of disease-associated mature type 1 FDCs, but the uniform electron dense deposit was sparse (Fig 4c). The expanded extracellular space was instead filled with many vesicular structures. These structures varied in size from 50–200 nm. Regressing FDCs as described above for controls were also found in scrapie-affected germinal centres. The occurrence of immature, mature and regressing FDCs in various genotypes in scrapie-affected and control sheep is summarised in Table 1. The PrP genotype did not affect nature of the FDC response to infection.

**Table 1:** Table summarising the occurrence of follicular dendritic cell types in control and scrapie-affected groups.

*FDC type*	control	Scrapie-infected
	PrP genotype-codon 136	
	*AA (n = 4)*	*AA, VV, VA (n = 20)*
**immature**	++	+
**mature**	+	+
**Disease-associated Mature type 1**	-	++
**Disease-associated Mature type 2**	-	++
**regressing**	+	+

(Score is based on frequency of follicles in which the FDC type predominates).

Three patterns of PrP^d^ immunolabelling were observed within lymphoid tissues from scrapie-affected sheep. Primarily, this labelling was confined to the germinal centres, but occasionally, PrP^d^ was present at sites distant from this.

**Figure 6:**
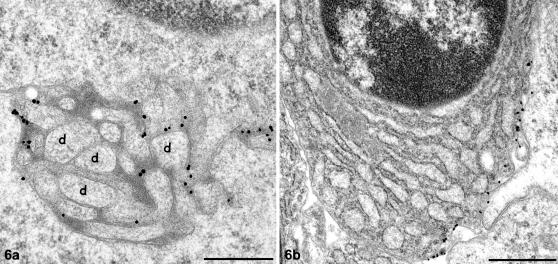
PrP^d^ on the plasmalemma of follicular dendritic cell (FDC) dendrites and a B cell. Scrapie-affected tonsil. PrP immunogold labelling. a. PrP^d^ is clearly limited to the plasmalemma of follicular dendritic cell (FDC) dendritic processes (d). Immunogold is not associated with the extracellular electron dense deposit held between FDC dendritic processes. Bar, 500 nm. b. PrP^d^ is also associated with the plasmalemma of B cells. Immunogold labelling is not present within the cytoplasm of the cell. Bar, 1 µm.

FDC-associated PrP^d^ immunolabelling was not abundant. Where present it was clearly limited to the plasmalemma of dendrites (Fig 6). No PrP^d^ immunolabelling was seen to co-localise with the electron dense deposit or vesicular structures within the extracellular space. Abundant labelling for immunoglobulins was associated with both the plasmalemma of FDCs dendrites and the accumulated electron dense deposit held between adjacent dendrites (Fig 5b), putatively in the form of immune complexes. Less mature FDCs with limited or absent accumulations of electron dense deposit, did not show immunoglobulin labelling.

Numerous terminally differentiated plasma cells with globulin-producing compartments were present within the germinal centres of scrapie-affected sheep and were unlabelled. However, PrP^d^ immunolabelling was also associated with the plasmalemma of a minority of plasmacytes characterised by distended rough endoplasmic reticulum. Labelling was restricted to small segments of the plasmalemma (Fig 6b).

**Figure 7:**
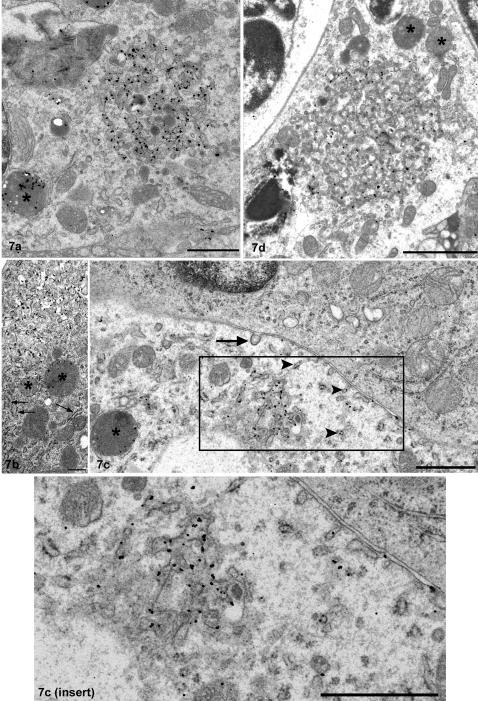
Intracytoplasmic tingible body macrophage (TBM)-associated immunolabelling. a. Scrapie-affected tonsil. PrP^d^ immunogold labelling. Immunogold labelling is present at the membrane of the random endoplasmic reticulum (ER) network within the cytoplasm of a tingible body macrophage (TBM). Immunogold labelling is also present within an adjacent lysosome (asterisk). Bar, 1 µm. b. Scrapie-affected tonsil. PrP^d^ immunogold labelling. Immunogold labelling is associated with the membrane of random ER networks within the cytoplasm of a TBM. Adjacent to these PrP^d^ labelled structures are areas of ER (arrows). Lysosomes are sparsely labelled (asterisk). Bar, 400 nm. c. (and insert). Scrapie-affected tonsil. PrP^d^ immunogold labelling. PrP^d^ immunolabelling is present on the plasma-membrane of a TBM. In addition, discreet vesicular structures contiguous with the plasma-membrane or within the cytoplasm of the TBM show immunogold labelling (arrowheads). Adjacent to these structures, a clathrin-coated pit is not labelled for PrP^d^ (arrow). A random network of ER is also immunolabelled. A lysosome shows PrP^d^ accumulation (asterisk). Bar, 1 µm. d. Scrapie-affected tonsil. Ubiquitin immunogold labelling. A random network of convoluted ER shows immunogold labelling of the membrane. Labelling is not present within the adjacent lysosomes (asterisks). Bar, 2 µm.

TBMs were numerous and present in both the light and dark zones of the germinal centres, and at sites distant from the follicle. TBMs contained abundant lysosomes and remnants of apoptotic cells. Frequently, within the follicle, large random networks of tubular structures were observed within the cytoplasm of these TBMs. TBM PrP^d^ immunolabelling was associated with both endosomes and lysosomes, and with the membrane of the fused tubular networks. PrP^d^ immunolabelled fused tubular networks were frequently observed immediately adjacent to PrP^d^ labelled lysosomes (Fig 7a), and in close proximity to patches of endoplasmic reticulum (ER) (Fig 7b). Occasionally, PrP^d^ was identified at the plasma-membrane and in association with single vesicular structures within the cytoplasm of the cell (Fig 7c). PrP^d^ was not associated with clathrin–coated pits (Fig 7c). PrP^d^ labelling of lysosomes and endosomes was restricted to more electron dense areas, most notably beneath the limiting membrane. Ubiquitin labelling was restricted to the membrane of the cytoplasmic tubular networks within TBMs (Fig 7d). Ubiquitin-labelled TBMs were observed both within the germinal centre and the mantle zone of the secondary follicles.

## Discussion

We have described species-specific morphological changes in FDC and TBMs of sheep scrapie-infected lymphoid tissues. This study further shows that accumulation of PrP^d^ is associated with the plasmalemmae of FDCs, a minority of mature B cells, and also with scrapie-related random fused tubular networks and lysosomes within TBMs. The first of these findings is consistent with those from scrapie-affected mice [18], [19].

FDC from normal sheep tonsil and lymph nodes can be classified into three overlapping morphological categories, attributed to immature, mature and regressing stages. Immature FDCs are also present in scrapie-infected lymph nodes. Consistent with previous descriptions [21], these immature FDCs, or their precursors, have limited or absent dendritic extensions with no surrounding electron dense deposit. Absence of labelling with anti-globulin antibodies, suggests that they have had little or no exposure to immune complexes. Upon antigenic stimulation, the capacity of immature FDCs to produce matrix elements is lost, while adhesion molecules, complement and specific surface antigens, are created [22]. Immune stimulation of primary follicles leads to the development of germinal centre-containing secondary follicles as a result of the deposition of antigen/antibody complexes on the surface of FDCs. This stimulates the development of extended dendritic networks associated with mature FDCs as demonstrated by immunoglobulin labelling [23]. Our results show that in regressing follicles of both scrapie affected and control sheep, immunoglobulin labelling is lacking and exosome-like structures are found in place of electron dense material between FDC dendrites.

Previous studies have demonstrated that antigen/antibody complexes are fixed in the extracellular space adjacent to FDC membranes via the antibody attachment to FDC receptor molecules C3b and Fc [24]. Mature FDCs present within lymph nodes of uninfected sheep have well developed dendritic extensions and small focal accumulations of electron dense deposit, which label for immunoglobulins consistent with exposure to, and subsequent accumulation of, immune complexes. The extracellular space around mature hypertrophic FDCs present within the germinal centres of lymph nodes of scrapie affected sheep consistently show a considerable excess of electron dense material when compared with controls. Because of its location, its similarities in density to that of immune complex material in uninfected animals [25], and the presence of immunoglobulins, we suggest that the excess of electron dense deposit correlates with abnormal accumulation of immunoglobulin.

The morphological changes of scrapie-infected FDC dendrites and accumulation of excess electron-dense material, suggest that the mechanisms of immune complex trapping is altered following scrapie infection. The electron dense space between dendritic profiles approximates to 1 micron, but antigen/antibody complex receptors confined to the plasma-membrane could retain complexes only to a distance of approximately 0.15 nanometers (nm) from it [18]. Therefore, not all of the presumed immune complexes can be trapped by receptors confined to the FDC plasmalemma. One possible explanation for the magnitude of antigen/antibody trapping is that primary FDC-associated immune complex may be capable of forming a large aggregate in association with complement, which is then capable of trapping additional immune complex.

**Figure 8:**
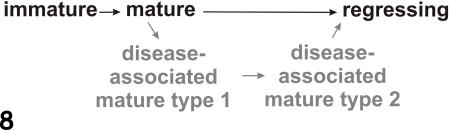
Diagram showing the maturation of normal and scrapie-affected follicular dendritic cells (FDCs). Following antigenic stimulation, immature FDCs, present in both normal and scrapie-affected animals, develop into a mature form of FDC. In scrapie–affected animals (grey), mature FDCs develop to form type disease-associated mature type 1 and subsequently disease-associated mature type 2 FDCs. We suggest the regression of normal mature and disease-associated mature type 2 FDCs results in the appearance of regressing FDCs within the germinal centre.

Sparse vesicles are associated with the extracellular space between FDC dendrites in uninfected animals (regressing FDCs), but numerous vesicles may be present in scrapie-affected tissues (disease-associated mature type 2 FDCs). Vesicles are 50–200 nm in diameter corresponding in size with exosomes; small membrane vesicles secreted by many cells as a result of endosomes or lysosomes fusing with the plasma-membrane. Vesicles at the surface of FDCs contain MHC-II molecules, but they do not synthesise them, suggesting that they are passively acquired from donor cells in the form of plasma membrane fragments, or exosomes, shed by B cells [26], [27]. In vitro binding experiments demonstrate that exosomes secreted by B cells specifically bind to FDCs. Trapped immune complexes serve to periodically restimulate B cells, and this active process results in deposition of B cell-derived exosomes that facilitate recruitment of corresponding B and T cells [26]. As the FDC matures, its capacity to trap immune complexes deteriorates [28], [29], and this, we propose, may result in abundant trapping of exosomes alone. Morphologically similar regressing FDCs are present in both control and scrapie-affected tissues. We suggest that the scrapie-affected FDC reaches a stage of regression identical to that of controls, at which point it loses its capacity to trap immune complexes and retain exosomes-producing B cells. Although the life span of exosomes remains unclear, it seems possible that due to the scavenging nature of TBMs, at least a proportion will be digested in this manner. The hypothesised maturation process of FDCs is illustrated in Figure 8.

PrP^d^ was present at the cell membrane of a small proportion of B cells. Survival of B cells within the germinal centre depends on whether their surface immunoglobulins are bound to antigens retained by the FDC. Specific antigens will bind to B cells in the form of immune complexes via the cell surface immunoglobulin receptor. During this process, we hypothesise that PrP^d^, present at the plasmalemma of the FDC may be passively transferred to the B cell plasmalemma. Were this not so, PrP^d^ would be processed and presented in a similar manner to antigenic proteins in association with MHC-II molecules. As such it would not be recognisable by the PrP antibody, nor would it be in an organised linear manner on the plasmalemma of the cell.

**Figure 9:**
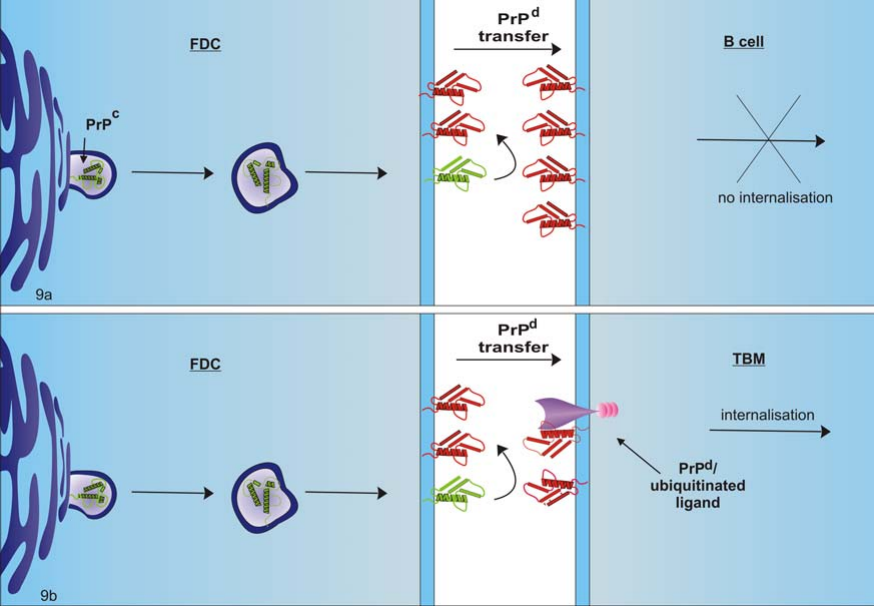
Diagram of the proposed mechanisms of PrP^d^ transfer and internalisation. a. Follicular dendritic cell (FDC)/B cell interactions. PrP^c^ is converted to PrP^d^ at the plasmalemma of the FDC. The molecule is passively transferred to the B cell during immune complex stimulation (possibly due to complex linkage), where it remains on the plasmalemma. FDCs do not re-internalise PrP^d^. b. FDC/tingible body macrophage (TBM) interactions. PrP^d^ may also be transferred from FDCs to TBMs where it is internalised after interaction with a trans-membrane ligand via a ubiquitin-mediated endocytic pathway and resulting in the accumulation of PrP^d^ at the membrane of the endoplasmic reticulum.

Pulse-chase experiments were the first to suggest that the transformation of normal to abnormal PrP may take place at the cell surface or at a later stage in the cell cycle [30]. In brain, PrP^d^ also accumulates on the cell surface [31], and may be transferred between cells *in vivo* (unpublished observations) and *in vitro* [32]. Similarly, TG3 PrP^0/0^ mice, which express PrP only on astrocytes but not neurons, show PrP^d^ accumulation and associated pathology on the non PrP-expressing neurons [33]. We suggest that PrP^d^ first accumulates at the plasmalemma of FDCs where it is then either transferred to adjacent cells to be retained on the plasma-membrane as it the case for B cells. Although exosomes transfer infectivity between cultured cells [34], we can see no evidence that exosomes in germinal centres are PrP^d^ positive. A mechanism for this transfer process is represented in Figure 9.

**Figure 10:**
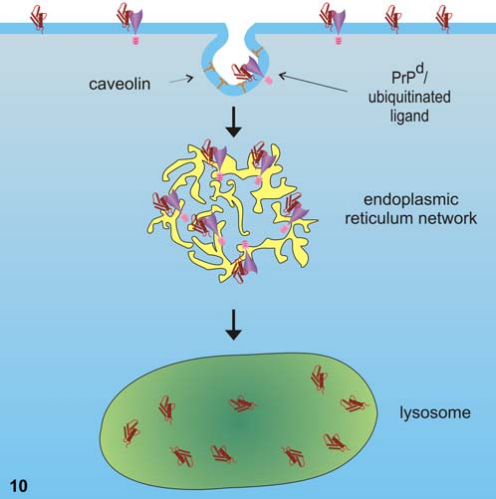
Diagram showing the process of internalisation of PrP^d^. More detailed schematic diagram showing a possible internalisation mechanism of PrP^d^ in tingible body macrophages (TBMs). PrP^d^ is only internalised into TBMs by the process of caveolin-mediated endocytosis. Once PrP^d^ reaches the fused random ER networks, it is then transported to lysosomes.

The primary function of TBMs is to phagocytose B cells that fail to produce viable immunoglobulins by affinity selection. TBMs can also ingest material from the extracellular space via clathrin or caveolin mediated endocytosis. Uptake of extracellular molecules is specifically mediated via receptors in caveolae [35]. Once internalised, caveolae travel to caveosomes where the ligands or membrane constituents may reside or be transported to the golgi or the ER. Random tubular networks, similar to those identified within TBMs in this study, were originally observed in simian virus 40 (SV40) infected cells. Such networks are extensions of the ER, formed following caveolin–mediated internalisation of certain ligands including SV40 and autocrine motility factor [36], [37], [38]. Alteration to the normal polygonal network of ER occurs following extension of the ER membrane and the subsequent absence of concomitant organising membrane proteins, leading to the collapse of the rigidly organised structure into the random arrangement [39]. In lymph nodes of scrapie affected sheep, inferred caveolin–mediated endocytosis results in the presence of abundant PrP^d^ in association with the membranes of random fused tubular extensions of the ER (Fig 7). This suggests that PrP^d^ may complex with other proteins to alter the normal endocytosis pathway and interfere with organising membrane proteins of the ER, perhaps also leading to an abnormal ER-lysosomal degradation pathway as depicted in Figure 10.

The immunohistochemical and ultrastructural alterations in the brains of the same sheep examined will be reported in a separate study. In brain, PrP^d^ and ubiquitin are associated with lysosomes and with abnormal clathrin coated pits and vesicles at neuronal and dendritic plasmalemmae [unpublished observations,40]. Thus, in the same scrapie infected sheep, PrP^d^ is internalised via ubiquitin-initiated clathrin endocytosis in neurons, while ubiquitin-initiated putative caveolin-mediated endocytosis occurs within lymphoreticular tissues. As normal PrP is not a trans-membrane protein, it must complex with an adjacent trans-membrane receptor ligand to be ubiquitinated and internalised [41]. Because different intracellular endocytosis mechanisms were observed at the cell surface of the same scrapie infected sheep, the molecule(s) interacting with PrP^d^ at the cell surface are likely to be cell and not strain dependent.

As ER-associated PrP^d^ has not been subjected to hydrolytic digestion, one would assume full length PrP^d^ will be present. We therefore further propose that the PrP^d^ labelled ER networks within TBMs correlate with the single intracellular granules of PrP^d^ labelling observed following application of extreme N terminus antibody BG4 [20].

In conclusion, several morphological changes are present in natural sheep scrapie-infected lymphoid tissue. In contrast to the dogma that scrapie does not functionally affect non-CNS tissues, we show that scrapie infection induces PrP^d^ related changes in the maturation of FDCs and probable immune complex trapping and processing. TBMs demonstrate a disease-related alteration to the normal caveolin-mediated endocytic mechanism and subsequent ER formation which differs from that of neurons in the same sheep.

## Materials and Methods

Ten sheep naturally infected and with clinical signs of scrapie, and four uninfected control animals from New Zealand were perfused via the carotid artery under deep surgical anaesthesia with 4% paraformaldehyde, 0.1% glutaraldehyde in 0.1 M phosphate buffer. All sheep were taken from susceptible PrP genotypes AA^136^ (n = 8), VV^136^ (n = 2) and VA^136^ (n = 4), (Table 1). Submandibular lymph nodes (SMLN) and tonsils were removed and immersed in fresh fixative. A further ten sheep (AA^136^) which were naturally infected with scrapie were killed, Mesenteric Lymph nodes (MLN) removed and immersion fixed in 4% paraformaldehyde. One millimetre thick slices of the lymph nodes or tonsils were taken for paraffin wax embedding and for electron microscopy. Tissues selected for electron microscopy were further trimmed into 1 mm^3^ blocks, post fixed in osmium tetroxide and embedded in araldite.

### Light Microscopy procedure–Wax

The light microscopical immunohistochemical procedure was used as described previously [42]. The monoclonal rat antibody R145 (VLA, Weybridge, UK), which recognises a C terminal epitope, and the monoclonal mouse antibody BG4 (Institute of Animal Health, Compton, UK), which recognises a proximal N terminal epitope, were applied over-night at 27°C, both at a dilution of 1∶4000 in incubation buffer. Protease K resistance of PrP was not tested, therefore the term PrP^d^ will be used to indicate all disease-specific PrP accumulations.

### Light Microscopy procedure–Resin

As described previously [19], the avidin-biotin complex immunohistochemical staining method using various anti-PrP antibodies specific to the N or C terminus of the PrP molecule, was applied to the etched and pre-treated sections. C terminus anti-PrP antibodies 523.7 (J. Langeveld, ID–Lelystad, Netherlands), R486 (R. Jackman, VLA, Weybridge, UK), F99 (K. O'Rourke, Washington State University, Washington, USA) and Bar224 (SPI-bio, Montigny le Bretonneux, France) gave substantial labelling of tissues embedded in resin from scrapie-affected animals. Of these, the polyclonal antibody 523.7 at a dilution of 1∶2000 in incubation buffer was found to give the most consistent labelling and was used routinely for all semi-thin resin and sub-cellular studies. Selected blocks with appropriate immunolabelled areas were then taken for sub-cellular studies. Again, the antibody used does not distinguish between the protease-sensitive and protease-resistant isoforms of PrP in biochemical extracts, however, the method employed in the study of TSE pathology in resin embedded tissues does not show any PrP labelling in control tissues. PrP detected in clinically affected sheep is therefore by definition, disease associated.

Sections were also immunolabelled with Zymed anti-IgG antibody at a 1∶100 dilution, or Dako anti-Ubiquitin antibody at a 1∶800 dilution using the same immunolabelling procedure with the omission of the formic acid stage [18].

### Ultrastructural immunohistochemical methods

65 nm sections were taken from resin blocks previously found to show PrP^d^ labelling and immunolabelled as described previously [19]. Primary antibody 523.7 at a 1∶250 dilution in incubation buffer, or pre-immune serum were used routinely. The above technique was carried out using anti-ubiquitin antibody at a dilution of 1∶50, and anti IgG antibody at a dilution of 1∶75. No formic acid step was required.

At least 5 follicles were studied from all LRS tissues collected.
